# A comprehensive review and update on human fertility cryopreservation methods and tools

**DOI:** 10.3389/fvets.2023.1151254

**Published:** 2023-04-18

**Authors:** Sevastiani Antonouli, Valentina Di Nisio, Christina Messini, Alexandros Daponte, Singh Rajender, George Anifandis

**Affiliations:** ^1^Department of Clinical Chemistry, Faculty of Medicine, University of Ioannina, Ioannina, Greece; ^2^Division of Obstetrics and Gynecology, Department of Clinical Science, Intervention and Technology, Karolinska Institutet and Karolinska University Hospital, Stockholm, Sweden; ^3^Department of Obstetrics and Gynaecology, Faculty of Medicine, School of Health Sciences, University of Thessaly, Larisa, Greece; ^4^Division of Endocrinology, Central Drug Research Institute, Lucknow, India

**Keywords:** fertility preservation, cryopreservation, gametes, embryo, gonadal tissue

## Abstract

The broad conceptualization of fertility preservation and restoration has become already a major concern in the modern western world since a large number of individuals often face it in the everyday life. Driven by different health conditions and/or social reasons, a variety of patients currently rely on routinely and non-routinely applied assisted reproductive technologies, and mostly on the possibility to cryopreserve gametes and/or gonadal tissues for expanding their reproductive lifespan. This review embraces the data present in human-focused literature regarding the up-to-date methodologies and tools contemporarily applied in IVF laboratories' clinical setting of the oocyte, sperm, and embryo cryopreservation and explores the latest news and issues related to the optimization of methods used in ovarian and testicular tissue cryopreservation.

## Routinely applied assisted reproductive technologies for fertility preservation

**Figure 1:**
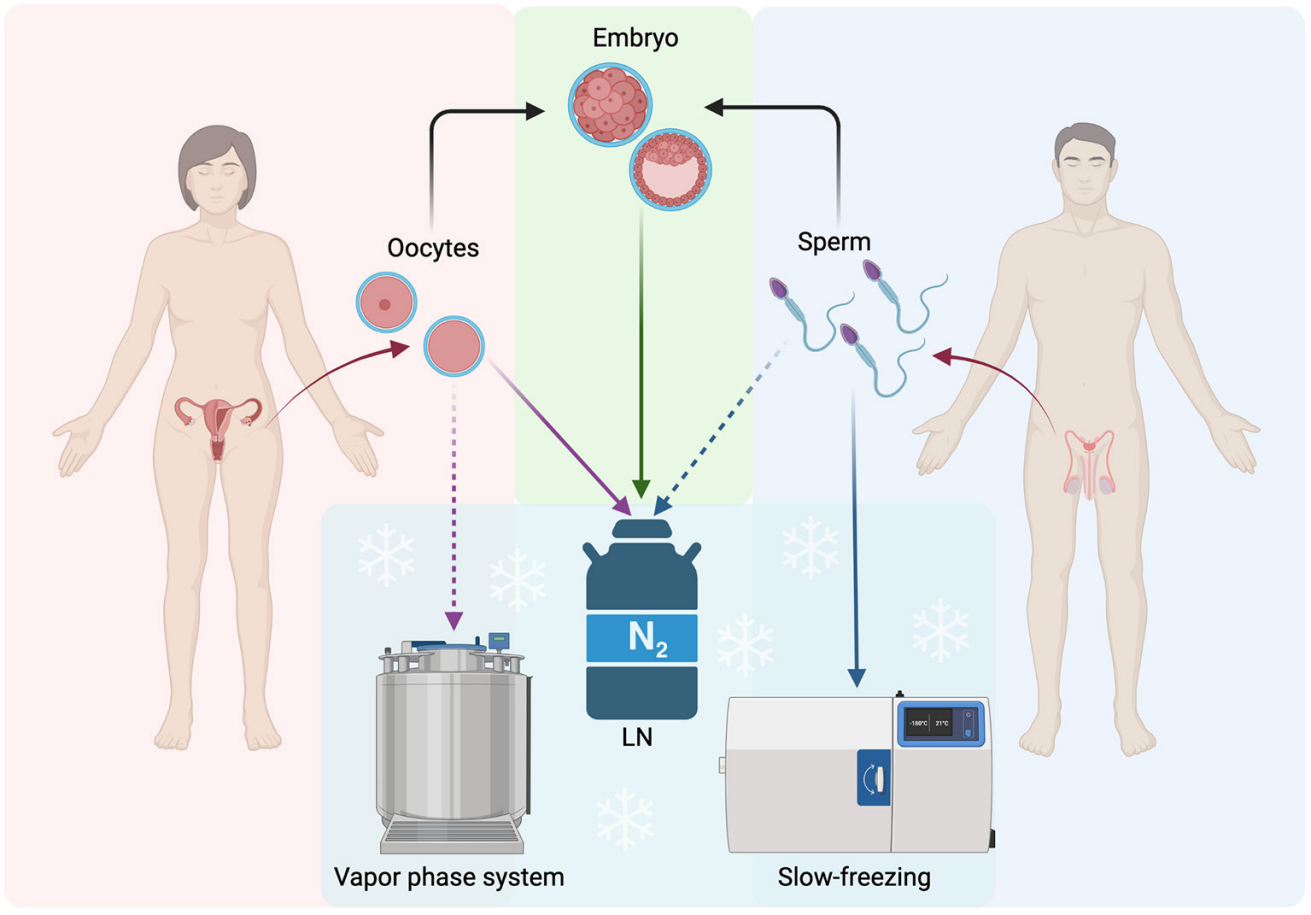
Schematic representation of the main routinely applied procedures for FP: gametes and embryos. LN, Liquid Nitrogen; Solid lines, often used procedure; Dashed lines, not often used procedures. The figure was realized with BioRender.

Increasing evidence demonstrates a global reduction in fertility rates. Unfortunately, infertility now is rising with ~48 million couples and 186 million individuals experiencing infertility or pregnancy failure despite people having frequent and unprotected sexual intercourse (1). This alarming evidence, in combination with the fact that the *in vitro* fertilization (IVF) success rate is less than the IVF failure rate, may cause “social” (mainly age-related) or medical concerns, such as the potential loss of fertility (2). Under these circumstances, fertility preservation (FP) is considered an option or a necessity. In 2018, the ASRM practice committee accepted social egg freezing as ethically permissible and named it “planned oocyte cryopreservation” (3). For all individuals, cryopreservation of gametes, embryos, and gonadal tissues/cells is the only way to achieve fertility in the future. Performing various cryopreservation programs, either freezing mature or immature oocytes may increase the chances of desired future pregnancies, especially in women of reproductive age that have been diagnosed with cancer and prior to subjection to any toxic chemo-radiotherapy treatments. Moreover, women who prefer oocyte cryopreservation instead of embryo cryopreservation and undergo an IVF program, and women who wish to preserve their ability for a future chance to have a baby for personal (“social”) or medical reasons. Medical reasons include mainly sickle cell anemia, severe endometriosis, diminished ovarian reserve, autoimmune diseases, and ovarian insufficiency risk due to genetic conditions such as the Fragile X premutation, and Turner syndrome (4). Other reasons are the X chromosome deletion and patients that have undergone gender diversity as transgender with affected fertility status (5). Practically, all the concerns regarding embryo cryopreservation, such as ethical, legal, and religious can be challenged by the oocyte cryobanking choice. At present, cryopreservation programs of sperm cells are in operation for supporting FP in men. Semen cryopreservation is one of the main choices where male fertility is compromised, such as for cancer patients who undergo chemo-radiotherapy treatments or vasectomy, HIV-positive, men undergoing gender reassignment since feminizing hormone therapy and orchiectomies can affect their fertility, and last for those with “social” concerns (6). In contrast to gamete cryopreservation, the embryo cryopreservation program is adjustable for having another attempt, after a negative IVF outcome, where spare embryos that have been cryopreserved can be used in a subsequent frozen embryo transfer cycle without repetition of ovarian stimulation and oocyte retrieval. Alternatively to oocyte freezing, embryo freezing can also offer FP in women diagnosed with breast cancer, as it is known that women with breast cancer are subjected to gonadotoxic treatments that may affect their fertility (7, 8). The performance of gametes or embryo freezing, and storage takes place in order to exchange them from donors to recipients after participation in donor programs following eligibility-approved criteria (9–11). Indeed, gamete and embryo cryopreservation are the main implemented practices and well-established techniques for FP, which are widely applied daily in clinics worldwide (Figure 1). A short history of both gamete and embryo cryopreservation, as well as clinical and technical improvements, are indicated in Table 1.

**Table 1:** History of the improvement of oocyte, sperm and embryo cryopreservation.

**Year**	**Achievements**	**Reference**
**Oocyte**		
1986	First pregnancy after human oocyte cryopreservation	(12)
1990–1996	Difficulties of oocyte crypreservation–slow freezing	(13, 14)
1997	ICSI implemented with slow frozen human oocytes	(15)
1999	First birth from vitrified oocytes	(16)
2005	First application of Cryotop method on human oocytes	(17)
2006	Oocyte cryopreservation as adjunct to conventional IVF, but still experimental	(18)
2010	Comparable delivery rate between fresh and cryopreserved oocytes	(19)
2011-present	Advances in cryodevices and artificial intelligence application	(20, 21)
**Embryo**		
1983–1984	First birth from slow frozen human embryo	(22, 23)
1998	Successful early cleavage of human embryos to the two-cell stage after ICSI	(24)
1999	First application of CryoLoop for embryo vitrification	(25)
2001	First human pregnancy and delivery of a baby as a result of blastocyst vitrification	(26)
2014	First trial of Gavi semi-automated closed system on human blastocyst	(27)
2015–present	Advances in artificial intelligence application	(21)
**Sperm**		
1949	First successful use of cryoprotectant glycerol in human sperm	(28)
1953–1954	First births following the use of human cryopreserved sperm	(29, 30)
1962	Preservation of human sperm in liquid nitrogen vapor	(31)
1964	First sperm bank in the world (Iowa and Tokyo)	(32)
1995	First birth from frozen epididymal sperm	(33)
1996	First birth from frozen testicular sperm	(34)
1990–2015	Optimization of protocols	(35–37)
2017	Novel micro-straw for small number of human spermatozoon	(38)
2018–present	Advances in artificial intelligence application	(21)

### The current state of human gametes and embryo cryopreservation

#### Oocytes and embryos

The most applied method of fertility preservation is oocyte cryopreservation and compared to embryo cryopreservation it is technically more challenging since the high-water content is risky for causing cryoinjury (11, 39). Over the last decade, embryo cryopreservation technology has been proven to be safe and effective (40).

Currently, in most embryological laboratories, the traditional methods of freezing and thawing of both human oocytes and embryos have been replaced by vitrification/warming protocols. Mounting evidence has shown the superiority of vitrification/warming over the slow freezing (SF)/thawing protocols, in terms of both the embryological and clinical outcomes (41–43). Vitrification is currently recommended for freezing oocytes and embryos as it has shown remarkably increased live birth rates (LBRs) (44, 45). Vitrification as well as SF, based on standard cryobiology principles, should ensure the accuracy and the success of the method with a minimal negative effect on the quality of the cell during the process of SF/vitrification and thawing/warming. To achieve successful vitrification in IVF it is required the control of three parameters, the concentration of cryoprotectant agents (CPA; viscosity), rapid cooling and warming rates, and media volume for preventing the intracellular crystallization of water (46). Cell stress during cryopreservation on cell/embryo structure is mainly coming from the direct effect of the cooling temperatures. For example, the hardening of zona pellucida due to premature exocytosis of cortical granules, the swelling of mitochondria in oocytes, and, the absence of tight junctions in embryos are some of the ultrastructural cryo damages that have been reported (47, 48). In addition, physical changes in regard to ice formation compromise the viability of the gametes/embryos since supercooled straws can cause various injuries during the ice nucleation phase or “seeding” (49). Noteworthy, cryopreservation indirectly depends on the quality of cryopreserved gametes, which in turn depends on the response to ovarian stimulation treatment (poor, hyper and normo-responders) and the quality of the sperm sample (poly, normo, oligo and azoo-spermia). Embryos coming from gametes of low quality may worsen further after thawing (50, 51).

The current application of ultra-rapid-freezing vitrification procedures involves the exposure of oocytes or embryos to little volumes of high-concentration CPAs (4–8 mol/L) at a very short time (avoiding chemical toxicity by the high concentrations of the cryoprotectants) followed by plunging the straw to liquid nitrogen (LN), in order to achieve solidification (52). During the process, the high osmolarity of the solutions used causes rapid dehydration and the cells are mainly dehydrated prior to freezing. SF dehydration initiates at the equilibration and continues up to −35°C. Afterward, it is taking place the submersion into LN quickly and solidification, so that the remaining intracellular water does not have time for the formation of ice crystals. In a brief time, <2 s, cells are transitioned from −35°C to −196°C, resulting in extremely fast rates of cooling (>10,000°C/min) (53).

The high CPA concentrations used in vitrification are linked with the risk of toxicity in cells (54). Recommendations nowadays are to mix different CPAs in order to avoid the potential toxic risk. The reason is that the combination of CPAs enables the reduction of individual components below their toxic threshold and, reduces to the minimum the time of exposure of oocytes/embryos to the solution (54). To date, the most common freezing solutions used are composed of permeating (e.g., ethylene glycol (EG), glycerol (G), dimethylsulfoxide (DMSO), propylene glycol, acetamide; >4 M) and non-permeating (e.g., sucrose, trehalose; >0.5 M) agents. The most applied protocol for both oocytes and embryos includes the combination of 15% DMSO, 15% EG, and 0.5 M sucrose at the minimum volume of ≤1 μL (17). A recent study showed that in the most used CPAs combination (DMSO and sucrose), while DMSO reduces the solute concentration, sucrose higher proportion has a direct effect on rising the Tg value, therefore increasing the sample safe storage temperature (55). Thus, it is needed to expand the knowledge of each CPAs thermodynamics, in order to find the optimal CPA combination for appropriate vitrification. Macromolecules, such as polyEG, ficoll, or polyvinylpyrrolidone that are used as supplements in the vitrification medium had been found to support vitrification with lower concentrations of CPAs. By further increasing the cooling rate (>10,000°C/min), which is needed for successful oocyte/embryo vitrification, the final volume of the vitrification microdroplet proved to be dramatically reduced, even to 0.1 μL (56).

Regarding oocyte vitrification, several rapid-cooling solutions and protocols have been developed over the years. Variations of the DMSO-based protocol were first introduced in 1998. Except for the DMSO-based protocol, it was developed a vitrification system consisting of a phosphate-buffered medium supplemented with 20% human serum albumin (HSA), and G and/or EG in increasing concentrations (57). For human oocyte and embryo vitrification, both systems are well implemented and represent current and best viable options differencing mainly in the presence or the absence of DMSO. For example, the absence of DMSO allows slower cooling rates, larger volumes of microdroplets, and different carriers. The first IVF cycles with vitrified oocytes have been reported (45), offering also in selected patients a “freeze-all” cycle by freezing all retrieved/collected MII oocytes as an option for fertility preservation (58).

Similarly, identical or slightly modified protocols are used for the rapid-cooling vitrification of cleavage-stage embryos (day 3) and blastocysts (day 5–7). The embryo transfer of frozen cleavage-stage embryos or blastocysts appears to have no difference in pregnancy outcomes (59). Artificial shrinkage of day-5–7 blastocysts has been shown to cause less cryoinjury during freezing/warming (60). Improved survival rates of hatching blastocysts have been found after puncturing manually the trophectoderm with a needle or laser. Notably, in 2014 Parmegiani and colleagues published a pilot study, suggesting a “universal warming protocol” (extracellular CPAs 1–0.5 M) as an efficient protocol for warming (61). Later on, in 2017 they applied this protocol on commercial brand kits (Kitazato and Sega kits containing trehalose and sucrose, respectively), proposing that a combination of different kits is efficient for the vitrification/thawing of embryos (62). Moreover, in regard to embryo vitrification and particularly in re-vitrification, interesting results have been found in the re-vitrification of 8 cells and blastocysts. Indeed, the twice vitrification/warming embryos demonstrated different effects on embryo developmental potential, as re-vitrification at the blastocyst stage followed by previous vitrification at the 8-cell stage decreased the delivery rate. Therefore, it has been recommended to be avoided. Twice-vitrified 8-cell stage achieved comparable pregnancy results to the once-vitrified embryos (63).

Nevertheless, vitrification as a process seems to have technical difficulty during its operation due to the highly concentrated, viscous, and minimal volume of solutions. Therefore, oocytes/embryos require fast handling (<1 min) and only well-trained embryologists can complete this task successfully. Besides, freezing/warming remains a time-consuming process (8–15 min). However, a current study proved that time reduction can be achieved by adding a two-minute dehydration protocol which aims at remaining the critical intracellular concentration needed for successful vitrification. The efficiency of the above protocol was verified by the post-warming survival rates and the ability of the warmed embryos to resume cell cytokinesis (64). Furthermore, in order to accomplish a successful vitrification protocol in combination with the technical difficulties, special carrier systems, open (direct contact of medium with LN) and closed (not direct contact) has been developed. Nowadays, more than 30 different carrier tools have been described, and half of them are commercially available (65). Open-pulled straws, such as Cryoloop and Cryotop were chronologically first introduced and, later on, closed systems were developed, as a more sterile and safe method compared to the open systems (66). Closed cryo devices include the Vitrisafe, the CryoTip, and the high-security vitrification kit (20). Other less usable straws include the Flexipet-denuding pipette, the electron microscopy copper, the gel loading tips, the Vitmaster, the Cryolock, the Cryoleaf, and the Hemi-straw system (56, 66, 67). Notably, when Cryotop was compared to CryoTip, it was found that the closed device demonstrated better survival rates, but the vitrified oocytes appeared with ooplasmic vacuolization, swollen mitochondria, and a high number of dispersed vesicles, possibly due to a less rapid decrease of the temperature in the closed carrier (68). In a recent study using the open Cryotop, and the Vitrolife system against the closed Rapid-i^®^ and the Kitasato system, the latest showed higher survival rates, but lower fertilization rates of the survived oocytes and no difference in the developmental competence compared to the open system (69). Based on the principal concept of cryo devices, in an attempt to minimize the volume of the vitrification solution for increasing cooling/warming rates, the novel device of the Kitasato System is currently under development in mouse embryo trials. This device is similar to the Cryotop device, differing in the possession of a porous membrane that absorbs excess vitrification solution around the embryos, in order to achieve more rapid cooling and warming rates, reaching 683,000°C and 612,000°C/min, respectively (70). Another new Argentinian device, the ZURE^®^ Vitri Carrier is now under construction and the scientific group focuses to obtain preliminary data on recovery and survival rates of vitrified and warmed oocytes (71). To date, current interest focuses on the possible effect of shipment and storage of gametes and blastocysts by comparing vapor and LN phases. The novel vapor phase TMRW platform, which was developed and improved by the use of artificial intelligence, was designed to allow safer handling and a digital chain of custody. Briefly, it consists of the Brooks BioStore III Cryo −190°C System, including a Chart MVE 1500 series tank with custom-designed CryoGrids that hold radiofrequency identification-enabled CryoBeacons serving as vessels for commercially available cryo devices and cryo straws. Evaluation of the data TMRW showed that this system does not have any deleterious effects on the survival rate of sperm, oocytes, and blastocysts. In addition, the storage of embryos in the TMRW vapor phase platform did not have any impact on the post-developmental potential of human blastocysts (72). Modern high-efficiency vapor phase tanks, such as the Chart MVE 1500, allow the storage of specimens in the vapor phase at temperatures below −150°C (73).

However, the Cryotop device, which was first described by Cobo et al. (74), is the most popular and best-selling micro-volume storage device (75). Nonetheless, there is a debate in relation to the sterilization of LN, because gametes and embryos are coming in direct contact with LN, increasing therefore the risk of cross-contamination. The open vitrification system is forbidden in several countries, including France, Belgium, Ireland, and the Czech Republic, despite the existing evidence that the risk is negligible and at a theoretical level (69, 76, 77). Indeed, the virus screening of culture medium and LN for HIV, hepatitis B, and hepatitis C viruses resulted in the absence of the respective viruses in all samples studied which were vitrified by using the open Cryotop device (78). No contamination of bacteria or fungi was observed in both open Cryotop and closed CryoTip carriers, after storing the human genetic material for 1–2 years, while the risk for cross-contamination for bacteria in animals was equal for both carriers (79). The potential contamination risk by the Zika virus was reported in the cryostorage of gametes and embryos, especially in semen. Nevertheless, there is not sufficient data regarding the virus survival in LN, although this probability has been reported by the British Fertility Society. Interestingly, a study published in 2021 comparing the efficacy and safety of closed High-Security Vitrification™ vs. open Cryotop devices, showed that the closed system had comparable survival, developmental, pregnancy, and implantation rates to the open system. According to this finding, the authors suggested the use of closed devices for eliminating the potential risk of viral contamination even during the presence of SARS-CoV-2 (80).

#### Sperm

The most valuable and popular method for male FP is the cryopreservation of spermatozoa, which is widely used in assisted reproductive technology programs. In contrast to oocyte and embryo cryopreservation, sperm freezing is much simpler due to the small volume of water inside the spermatozoal cells.

SF, rapid freezing, and vitrification are sperm cryopreservation methods, discovered during the performance of several cryo-experimental improvements (81, 82). SF was developed by Behrman and Sawada and includes progressive sperm cooling in two or three steps, either manually or automatically (37, 83). SF was replaced over time by rapid freezing and vitrification protocols since SF is time-consuming and when performed automatically requires an expensive machine as a programmable freezer (84, 85). Optimal sperm survival rates depend strictly on the usage of CPAs and the cryopreservation process itself. Both permeable (e.g., DMSO, G, EG, and 1,2-propanediol) and non-permeable CPAs (carbohydrates such as glucose, sucrose, and trehalose) are routinely used for sperm freezing. Permeable CPAs due to their lipophilic properties can easily cross the sperm cell membrane, something that is associated with increased cell toxicity. The non-permeable CPAs are of high molecular weight and do not cross the membrane, making them effective for increased freezing speeds, and promoting rapid cellular dehydration (86–88).

Conventional SF is performed by using permeable CPAs and is still the most commonly applied technique for sperm freezing (37). SF allows the preservation of a relatively large volume of sperm ejaculate (89) but during the process, almost 50% of spermatozoa are lost by cell lysis caused mainly by the formation of ice crystals. This compromises the semen quality after thawing (90). Indeed, the cooling rate plays a key role in evaluating the extent of sperm cryoinjury since basic morpho-functional abilities of the spermatozoa might have been affected. Thermal shock, formation of intracellular and extracellular ice crystals, changes in membrane permeability, cellular dehydration, and osmotic shock are major cryo damages that may occur during the addition or removal of CPAs (91). In contrast to SF, rapid cooling and vitrification are fast protocols that do not cause ice formation during the process, while the cooling rates reach 3,000°C/min (92, 93). Proposed firstly by Sherman, rapid cooling is performed by freezing sperm in LN vapor, requiring direct contact between the straws/vials and nitrogen vapors (8–10 min), followed by rapid immersion in LN at −196°C (94). At vitrification, as suggested by the WHO manual for sperm analysis (95), the sperm samples are directly plunged/dropped into LN, reaching ultra-rapid cooling rates at seconds and thus preventing any possible formation of ice crystals. In addition, the osmolarity of the media appears to play a crucial role in avoiding membrane damage. Besides the osmolarity, similar importance seems to play the temperature during the thawing/warming process. Sperm vitrification requires the removal of seminal plasma before starting the procedure, while in other sperm freezing techniques, semen removal can be done either before freezing or after thawing (82). Seminal plasma may contain cell leukocytes or other microorganisms with the ability to promote the production of reactive oxygen species (ROS). It was found that either the swim-up or the density gradient centrifugation sperm preparation method, may eliminate the presence of ROS and the possible damage to the spermatozoa (96, 97). Improved sperm recovery rates (in terms of sperm motility) were found in comparison to conventional SF, while the levels of DNA oxidation, and mitochondrial activity remained unchanged (98). In line with the previous conclusion, a recent meta-analysis found better sperm recovery rates with the vitrification method when compared to conventional SF techniques. A drawback of that meta-analysis was the limited number of studies performed with sperm vitrification (84).

As mentioned above, vitrification requires high cooling rates and elevated CPA concentrations in order to bypass the phase of ice crystal formation during glass solidification. Notably, the high permeable CPA concentrations cause damage to spermatozoa. At present, the most commonly used CPAs for sperm vitrification are the permeable CPAs (including DMSO, G, glycol, EG, and methanol), and non-permeable CPAs (including albumins, dextran, and egg yolk citrate) (99). Recent clinical trials investigating the role of various concentrations and mixtures of CPAs at low sperm amounts (at around 20 μL of a sperm suspension drop), are ongoing to optimize the ideal mixture, concentration, and volume (100). Indeed, the investigation is focused mainly on the development of a vitrification protocol CPA-free (101, 102). Isachenko and colleagues were the first who succeed in the development of human sperm vitrification CPA-free (103, 104). In the same line, Slabbert and colleagues performed sperm vitrification CPA-free, suggesting that the CPAs-free technology results in higher mitochondrial membrane potential and lower sperm DNA fragmentation post-thaw when compared to conventional SF (105). Similarly, the permeable CPAs-free vitrification was found to produce better sperm recovery rates, in terms of intact acrosomes, more viable spermatozoa, and of a lower percentage of sperm DNA fragmentation when compared to conventional SF (106).

Although 0.25 mol/L sucrose has been widely used for both rapid freezing and vitrification of human sperm (101, 105), the optimal concentration of trehalose is still under investigation and more trials with different trehalose concentrations are necessary to standardize the rapid freezing and vitrification method. Comparisons between of 0.1 mol/L trehalose and 0.25 mol/L sucrose demonstrated that the first resulted in enhanced sperm motility post-warming when samples were cryopreserved in a closed device system. Moreover, sperm preservation with 0.1 mol/L trehalose showed improved membrane integrity at 0 h post-thaw, while after 6 or 12 h, there were no significant improvements in comparison with sucrose (107). Current trials in optimizing trehalose concentration revealed that the concentration of 0.125 mol/L is more beneficial in comparison to 0.25 mol/L (108). Additionally, combinations of CPAs, such as human tubal fluid, sucrose, and butylhydroxytoluene have been also proposed (103, 109–111).

Over the past years, apart from the indications for different combinations of CPAs, or even the absence of them, it has been also performed analogous testing in various cryo devices. With respect to each device, different volumes/droplets have been tested, but never more than 0.5 ml. Indeed, both rapid cooling and vitrification depend on the volume of sperm suspension, while only small volumes of 1–30 μl were reported in either closed or open systems. The results of every investigation emphasize the need for better protocols following the aseptic cryopreservation (101). Another limitation of the vapor method freezing is that it is unable to control the cooling rate caused by the volatilization of LN (37). A study evaluating sperm motility, fertilization ability, and DNA integrity in a Cryoloop open system using both the vapor and vitrification protocol, showed that there were no differences between the methods tested within the same device (112). When it was evaluated both protocols using cryovials, sperm motility and DNA fragmentation levels were comparable (113). Recently, a study testing six different cryopreservation methods using the novel funnel-shaped device, revealed the superiority of vitrification with 0.3 M sucrose and 20% (v/v) dextran supplement. Moreover, this study revealed optimal results in both conventional SF and vitrification (100). The 0.5 mL straw for rapid freezing and the straw-in-straw for the vitrification system showed no significant differences regarding the percentage of post-thawing sperm DNA damage (114).

Similar to the cryo devices for oocyte and embryo cryopreservation, many also open systems have been developed for sperm vitrification. For example, the 5-mm copper loop, the cryoloop, the open-pulled straw, and the open-standard straw (112, 115). The potential risk of contamination is one of the major disadvantages when freezing semen samples with the open vitrification system due to direct exposure to LN (116). Using a closed carrier during the vitrification process is not always feasible. Moreover, mounting evidence reveals that the storage of sperm samples using the vapor protocol can decrease the risk of viral contamination (and cross-contamination) (117). Apart from the open systems, a variety of closed tools have been proposed and developed in the vitrification process and some of them are the straw-in-straw, the high-security vitrification straw, the Cryotip, the VitriSafe, the Cryopette, the cryptologic, the Rapid-i, the S3 system, and the S3 μS-VTF device (99). Regarding the ultra-rapid vitrification, in an attempt to freeze a small number of spermatozoa, a study suggested a novel approach based on the use of micro-straws (50–100 μl) instead of the traditional straws (0.25 and 0.5 ml) (118). As the micro straws are thin retaining very small cryo-volumes, the freezing rate is faster. These devices showed better sperm motility values after post-thawing maintaining simultaneously the morphology and sperm DNA integrity (38). Other carriers for cryopreserving oligospermic samples and microquantities have been developed, such as the empty zona pellucida, the Volvox globator spheres, the alginate beads, the agarose microspheres, the microdroplets, the straws, the mini-straws and, the open-pulled straws, cryoloop (37, 119). In 2020, a novel sperm tool, named SpermVD, was described as a carrier of high efficiency for freezing a small number of spermatozoa in low-volume droplets. This tool has the advantage of an almost 100% recovery rate post-thawing and eliminates the post-thawing time search for the recovery of the spermatozoa. Moreover, the results obtained by this tool were promising and in particular in men suffering from non-obstructive azoospermia (120). Nowadays, the development and optimization of new devices are based on the progress of 3-D printing technology. This technology makes feasible the production of such advanced freezing devices through complicated designing and printing methods, optimizing and standardizing the accuracy of sperm cryopreservation (121). Nevertheless, the majority of protocols for sperm vitrification are not standardized yet in order to be used in clinical routine. In this context, aseptic techniques can be applied, since the cytoplasmatic membrane of the spermatozoon is exposed to LN, making it more susceptible to the adhesion of microorganisms present in LN during the cryopreservation process. Unlike to the spermatozoon, the cytoplasmatic membrane of the oocytes and embryos is covered by the presence of zona pellucida. Besides, these protocols rarely attain satisfactory post-thawing sperm motility and viability values. Although the above evidence has been recently reported, a survival rate of more than 70% with a new approach (122). The vitrification process is considered more applicable for sperm samples with an extremely low number of spermatozoa, such as those retrieved after testicular surgical sperm extraction. In addition, the storage of vitrified sperm samples appears to have a significant role in cryopreservation strategy, bearing in mind their use in intrauterine insemination protocols.

## Not routinely applied assisted reproductive technologies for fertility preservation

**Figure 2:**
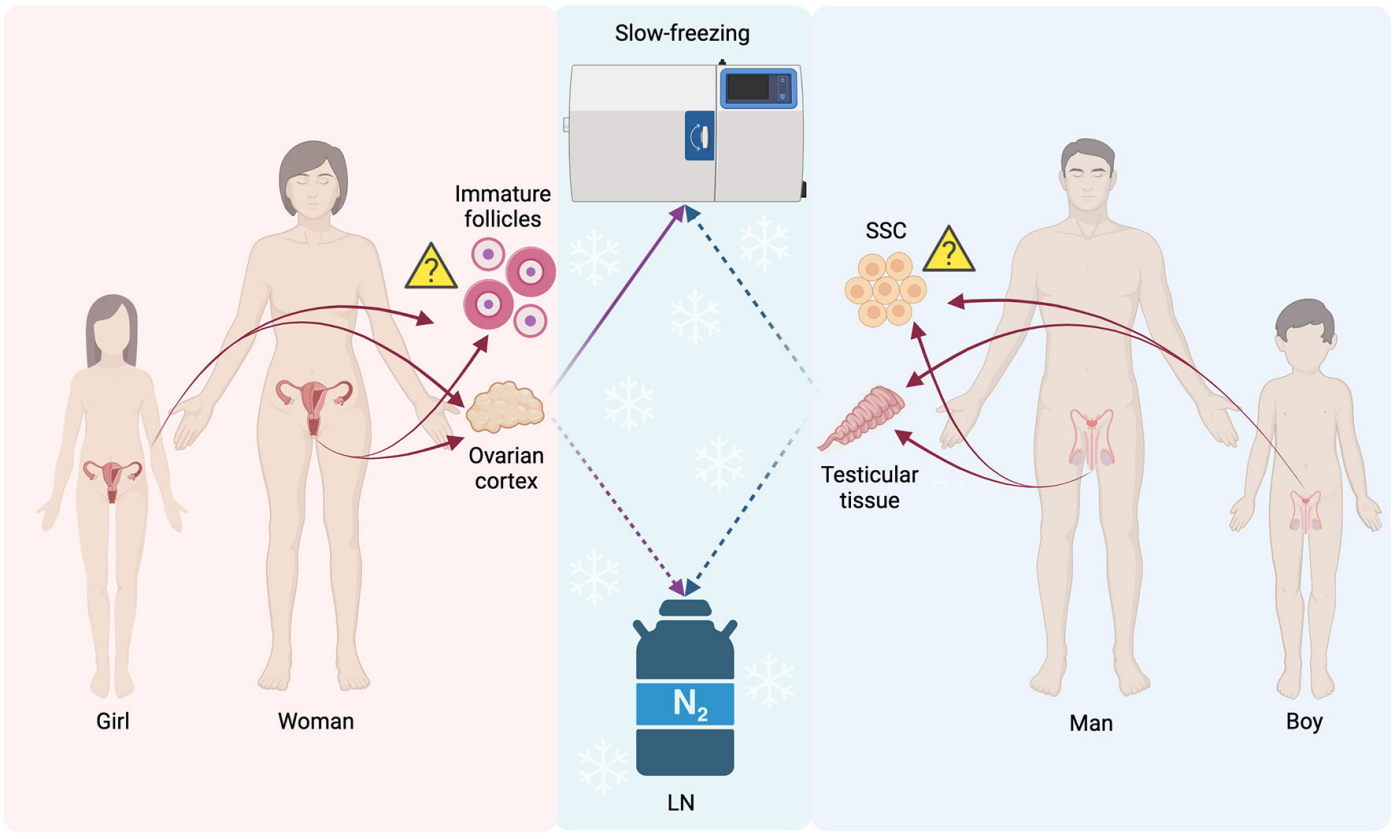
Schematic representation of the main not routinely applied procedures for FP: gonadal tissues, follicles, and spermatogonial stem cells. LN, Liquid Nitrogen; SSC, spermatogonial stem cells; Solid lines, often used procedure; Dashed lines, not often used procedures/experimental; Triangle, still undecided if feasible for FP purposes or not. The figure was realized with BioRender.

In contrast to patients that have the possibility to be subjected either to gamete or embryo cryopreservation procedures in order to preserve their fertility, there is a percentage of patients that cannot undergo the routinely applied processes of fertility preservation. As far as those patients are concerned, gonadal tissue cryopreservation has been indicated as an alternative aiming to safeguard their fertility. Ovarian tissue cryopreservation (OTC) is the only option for girls with cancer at the prepubertal stage since the potential risk of stimulation of estrogen-sensitive cancer can be bypassed (123, 124). Other patients that may benefit from the OTC programs are those having recurrent ovarian cysts, ovarian torsions, and autoimmune diseases as well as women of early reproductive age who wish to delay their menopause (defined as “social freezing”) or to avoid the syndrome of premature ovarian insufficiency. Patients with Turner syndrome or transgender men may also benefit from the OTC program (8, 124–126). The goal of OTC is to maintain the morpho-functional characteristics of the ovary providing in this way a huge supply of primordial follicles, which can be successfully used in the future, preserving their fertility. Systematic reviews in regard to the clinical outcome of ovarian transplantation range between a minimum of 42% and a maximum of 81%, denoting the clinical importance of using this fertility cryopreservation method (127, 128). Noteworthy, 130 healthy babies were born worldwide with the applied OTC (129) two women only worldwide have undergone transplantation with the OTC program which resulted in three live births after transplantation (130, 131). Testicular tissue cryopreservation (TTC) is recommended for boys who had been diagnosed with cancer at the prepubertal stage since 30% of the survivors (a cancer survival rate of 80%) appears to encounter azoospermic issues in the ambient future (132). TTC is indicated also for adolescents diagnosed with testicular cancer, leukemia, or Ewing sarcoma and who have an elevated risk to develop permanent sterility issues after gonadotoxic chemo- or radiotherapies (133). Other patients that may benefit from TTC programs are those having severe autoimmune diseases, genetic and congenital diseases such as those who suffer from the Klinefelter syndrome, and, other life-threatening non-malignant diseases such as drepanocytosis, thalassemia, idiopathic medulla aplasia, and the granulomatous disease. The same group involves patients who are subjected to gonadotoxic therapies, such as full-body radiotherapy, as well as prepubertal boys with a high risk to acquire infertility problems due to bone marrow transplantation (134). However, the clinical application of OTC and TTC is not well-established, and further research and improvements are needed regarding the protocols and the tools used for these occasions (Figure 2). Although improvements in OTC and TTC methods are essential, a major concern is raised in patients affected by oncological pathologies. In this category, it may be an increased risk of reintroducing malignant cells after tissue transplantation. This main drawback is specifically explicated at the end of the following sub-sections.

### Human gonadal tissue cryopreservation: an ongoing process

#### Ovarian tissue

Nowadays, the OTC programs for fertility preservation procedures are gaining ground in regard to clinical application, holding hope for all patients that couldn't have undergone routine FP programs (8, 129). OTC is applied solely to the ovarian cortex area. Specifically, a 1 mm area is surgically removed from the ovary and cryopreserved from a surface area that ranges from 2 × 2 to 5 × 5 mm (135). Thus, after collecting the cortical ovarian tissue, the somatic and germ cells are frozen with the ultimate scope to preserve early-stage follicles for either future autologous transplantation or *in vitro* oocyte maturation (136, 137). Autologous ovarian tissue transplantation (OTT) can be orthotopic, meaning that it will be reimplanted in the pelvic cavity or in the ovarian medulla. In a recent study, the ovarian function was restored with 90% success (124). When OTT is heterotopic, the biopsy may be reimplanted outside the peritoneal cavity (138). In comparison with other FP procedures, the OTC process is of increased complexity, owing to the presence of multiple and diverse cell populations. It is known that the presence of these cells may negatively affect the permeation of cryoprotectants, thus also affecting the survival of the follicles (81). In contrast to oocyte vitrification, OTC showed a decreased LBRs trend, but still, today remains the only promising choice for some of the aforenamed patients (124). Notwithstanding, both OTC LBRs and OTT outcomes, could be largely improved by ameliorating the revascularization of the reimplanted ovarian tissue with angiogenic and antiapoptotic factors (129). Interestingly, there is no global consensus about the cryopreservation protocol regarding OTC, even if several trials have been made to develop a whole ovary cryopreservation method (139, 140).

In this matter, two are the procedures for ovarian tissue freezing. The preferable one is the SF method. In this method of paramount importance is the choice of the permeable CPAs (propanediol, DMSO, or EG in a concentration of 1.5 M) in combination with the non-permeable sucrose (~0.1 M) (141). The main factors affecting the SF procedure are the different compositions of the cell types and the complicated extracellular matrix composition. Both these factors should be taken under serious consideration in order for cryoprotectants to gain access to the inner part of the tissue. Toward this, it is essential to separate the ovarian tissue into thin strips or small squares at the initiation of the procedure (135). In 1994, Gosden and colleagues developed for the first time a protocol for OTC. Based on this protocol all the subsequent protocols developed are deviating either in time or in the temperature of the CPAs (142). Briefly, after the equilibration of ovarian tissue with CPAs at 0°C, the tissue stripes are inserted in a programmable freezer where the temperature is gradually reduced to −7°C (for manual seeding) followed by another reduction of the temperature up to −140°C. Finally, the ovarian tissue stripes are cryopreserved and stored in LN for long-term storage (141). In late past years, many investigations have tried to improve the SF protocol, mainly focusing on the notion to reduce the formation of ice crystals that may damage the tissue. The long-time cooling of the ovarian cortex before OTC seems to decrease the translocation of phosphatidylserine in the tissue (143), but a recent application of this protocol found that 24 h cooling of human ovarian tissue at 5°C increased the viability of cells post-thawing (144). Moreover, the use of 20% DMSO concentration allowed a decrease in ice formation, however, it did not increase the survival of the follicles after xenograft (145). Interestingly, promising research has been conducted on sheep ovaries, but until now it was not tested on human ovaries (146), emphasizing once more the lack of evidence for a unanimous OTC protocol.

Despite the SF as a method that is currently the golden standard for OTC, much progress has been made in ovarian tissue vitrification (OTV), aiming to improve the survival of the tissue after warming (147). In Japan, two babies have been born by this method (148, 149). The vitrification procedure is mostly based on the balance between the ultrafast cooling rate and the high concentrations of CPAs. Bearing in mind that high concentrations of CPAs are toxic, then the combination of two or more CPAs seems to be essential because on one hand enhances the support of the tissue and on the other hand decreases the toxicity of the CPAs (150). The small volume during OTV appears to influence negatively the success of OTV. The absence almost of liquids and the difficulty to form ice crystals are attributed to the small volume during vitrification (149). In an attempt to avoid the larger volumes of CPAs, various tools have been described as for instance the solid surface (151), the medium droplets (152), the plastic straws (153), the open Cryo Type M, the closed CryoSheet device (154), and the silver closed vitrification system (155). Interestingly, the use of the stepped vitrification device with high DMSO concentrations resulted in reduced follicle membrane disintegration (156). Additionally, in an OTC protocol through the method of slush nitrogen vitrification which increases the cooling rates and reduces the so-called “Leidenfrost effect”, it was observed better results in terms of follicle ultrastructures, viability, and stromal cell integrity (157, 158).

Due to the lack of consensus regarding standard protocols for the OTC, there are still a few matters that make the FP process more challenging. Among them, follicle survival as well as the size of the ovarian cortex for re-transplantation are major issues that need to be addressed. Recently, Kristensen et al. proposed the vital dye-neutral red method as a quantitative approach for the evaluation of follicle survival after thawing (159). This study, conducted in a non-clinical setting, pointed out the great follicle survival (average of 84%) in ovarian tissue strips cryopreserved through the SF method. Nevertheless, the main issue remains unresolved, since it is impossible to transplant to the patient exactly the same ovarian strip which was intended for investigation. The size of the re-transplanted ovarian cortex is playing also a major role in FP procedures. Large cortical pieces demonstrate greater follicular activity (160, 161), while small pieces (of 1 mm^3^ approximately) show reduced follicle growth (162).

Other alternatives to OTT, whenever this procedure is not feasible (e.g., cancer patients, transgender women, or polycystic ovary syndrome patients), are the *in vitro* maturation (IVM), the maturation of small immature follicles, and the use of “artificial ovaries” (163). The follicles are extracted from the tissue and matured *in vitro* for obtaining MII oocytes for further assisted reproductive technologies processing (164, 165). Similarly, a multistep culture technique has been developed for the *in vitro* activation of follicles from the primordial to the antral follicle stage, followed by the retrieval of the oocyte for the subsequent IVM procedure (166, 167). Experimental studies of this technique on human primordial follicles showed promising results up to the retrieval of the MII-phase oocytes (168). Although those studies are in the early experimental phase, huge hopes rely on the creation of a biodegradable scaffold, the so-called “artificial ovary”, that support the growth and development of human follicles, either *in vitro* or after autologous transplantation (169, 170). Toward this, multiple types of scaffolds have been developed, such as fibrin-based (171–173), 3D printed microporous hydrogel (174) and decellularized ovaries (175–177). Most of them have been used for follicle culture in animals and humans followed by transplantation in sterilized mice with promising results. Indeed, primary results have been obtained in mouse models. The experimental achievement regarding the transplantation of artificial ovaries in ovariectomized mice may shed light on the path of using OTC for the purposes of FP, reducing also the potential risks for women (178).

Patients suffering from hematological diseases (such as leukemias), neuroblastoma, or Ewing's sarcomas should be handled with extra caution, because of the potential presence of cancer cells in the vasculature or in the soft tissue that will be cryopreserved. Much attention has been given to cases of cancer patients during OTT because in some cases there is a major risk to reintroduce malignant cells into the ovarian tissue (179). Immunohistochemistry, molecular analysis, and investigation of the ovarian biopsy after xenotransplantation in mice models have been applied clinically in order to detect the presence of residual malignant cells in the tissue. However, the aforementioned tests require the analysis of the ovarian tissue, making it impossible to transplant the same ovarian strip in the patient (180). A possible treatment of the ovarian tissue before transplantation is the so-called “ovarian purging”. This method involves the treatment of the tissue with inhibitors that affects exclusively the malignant cells, leaving untouched both follicles and stromal compartments (181). Several *ex vivo* studies on human cryopreserved ovarian tissues from patients diagnosed with leukemia showed that the treatment of the tissue with tumor-specific inhibitors, such as DARP in-toxin fusion proteins, Verteporfin, Everolimus, and Aurora kinase B/C inhibitor, successfully reduced the cancer cells without impairing the survival rate or the growth potential of early-stage follicles (182–185). Thus, the application of this method may diminish the risk of the reintroduction of tumor cells to the ovarian tissue that will be transplanted, thus enhancing the possibility of FP in cancer patients.

#### Testicular tissue

Although sperm cryopreservation appears to be the optimal choice for male FP, a percentage of men with infertility should preserve their fertility with different methods since they do not have spermatozoa in their ejaculate. These methods include the isolation of spermatogonial stem cells (SSC), testicular cell suspension (TCS), and testicular tissue cryopreservation (TTC) (134). Among these procedures, TTC is the preferable option since cryopreserves SSCs with the surrounding supportive microenvironment, allowing in this way the transplantation of only either the SSC or the whole tissue, which in turn allows performing *in vitro* spermatogenesis after thawing (186). To date, the methods that exist for TTC are the controlled and uncontrolled SF (CSF and USF, respectively). Last, the vitrification option is currently applied in animals but is still in an experimental phase in humans (187–189). As far as TTC is concerned, the SF method (particularly the CSF) is considered to be the golden standard, in terms of optimal tissue preservation and prevention of ice crystal formation (134, 189). Data regarding the TTC method showed significant differences between the DMSO-based medium for immature TTC and the G-base medium for mature TTC (190, 191). Similar to ovarian tissue, when performing TTC needs to be taken into consideration factors such as the presence of several different cell types, the CPAs permeation time, the temperature as well as the size of testicular tissue, which range from 1 to 9 mm^3^ (192, 193). While it has been described SF and vitrification methods regarding the survival rates of human SSC in xenografted tissues (134, 191, 194), it is obvious that additional studies are still necessary for improving testicular cryopreservation methods in order to be applied in clinical settings.

Although CSF is currently recognized as the most preferable and efficient method for TTC (195, 196), the scientific community did have not yet reached a consensus on a standard protocol between CSF and USF. Methodologically the difference relies on the control of the cooling rate. While CSF requires the use of a programmable freezer to reach a controlled cooling rate of around −8°C, followed by manual seeding, the USF method involves the use of an isopropyl alcohol container (generally the Mr. Frosty Freezing Container) placed overnight at a −80°C freezer with an uncontrolled cooling rate of around 1°C/min (187). Pioneer studies point to the use of combined culture media with DMSO favoring the permeable CPAs in combination with the non-permeable sucrose (195, 197–200). When CSF was performed in the presence of DMSO, studies evidence the better-cryopreserved ultrastructure and higher survival rates of Leydig and spermatogonial cells after a 12- and 24-day *ex vivo* culture (192, 198, 201). Additionally, the effect of Leibovitz L-15 in the culture medium when compared to phosphate-buffered saline showed no sign of tissue damage, neither in terms of morphological features nor in spermatogonial cell survival rate (202). Interestingly, CSF when was applied to TCS the results were comparable with the TTC, pointing out that TCS cryopreservation may have a potential role at a clinical application level. Nevertheless, further studies are required for the confirmation of these preliminary data (203, 204).

The vitrification of testicular tissue as an ultra-rapid freezing technique is still in an experimental stage, therefore is not applied for any FP procedures. Until today, only a few investigations have been conducted in humans for the development of a testicular tissue vitrification (TTV) protocol. The results were not so convincing in order to promote the use of TTV over the standard method of CSF. TTV was investigated in young-derived testicular tissue in which the tissue was directly plunged into LN within cryo straws, and compared to CSF followed by short-term culture (205) or long-term xenografting (194) after warming/thawing. In all experimental studies, when comparing the CSF and the TTV protocols, the authors denoted a similar morphology, by using hematoxylin-eosin, and a comparable presence of proliferative spermatogonial cells, by using the MAGE-A4 and Ki67 markers (194, 205). Concomitantly, Baert and colleagues explored two different approaches for TTV, solid-surface vitrification (SSV) and direct cover vitrification (DCV), and compared them with CSF and USF methods. In brief, SSV was performed by placing the tissue on aluminum floaters partially immersed in LN, while in the DCV method, the tissue is placed in a cryovial and plunged into LN (187). Despite the fact that in the SSV method, a minimum of spermatogonial-cell ultrastructural damages was found, the number of SSCs recovered after thawing was drastically reduced in comparison to the CSF protocol. These results indicate that vitrification is the best cryopreservation protocol for SSCs but only in the cells that are able to survive the ultra-rapid freezing process. Finally, the scientific background regarding vitrification at TCSs is relatively poor, therefore it cannot be considered as a valuable option besides the TTC method. Only two papers have been published, in which they show a higher survival rate of vitrified TCS in comparison to the CSF one (206, 207). Besides, both publications evaluate exclusively the quality of cell integrity and the survival rate, but not their functionality. Further studies are needed also to identify the ideal TTC procedure for FP protocols in order to use it in clinical practice.

Due to the lack of a standard TTC protocol, it is worth mentioning the different devices that may help during cryopreservation, especially in the TTV methods. Among the opened options, the open-pulled straws have been tested on TTV. The results were very encouraging as far as integrity and functionality are concerned (194), although the open device jeopardize the tissue due to the possible risk of contamination and cross-contamination by the LN of the tank (previously described in this review). The issue of contamination and cross-contamination is not encountered when closed devices (such as cryovials) are used, or during the application of SSV. These methods are extensively studied in small and large animals (208–211) but lack an optimization regarding human testicular tissue (187).

The main drawback of TTC and testicular tissue transplantation (TTT) in cancer patients is the potential risk of reintroducing malignant tumor cells to the implanted tissue. This issue has been given much attention in both TCS and testicular tissue samples (212–214), although in some human experimental settings the authors were able to sort malignant leukemic cells out of the TCS (215, 216). Altogether these results raise concerns about the safety of TTT in cancer patients, highlighting the need for further studies.

## Accessibility considerations to fertility preservation: recent guidelines and programs for patients' recruitment

The exponential need for FP in the whole gamma of patients mentioned in the text makes the need of presenting detailed guidelines for the standardization of protocols in order to be applied to IVF clinics either inside the European context or outside. These rules should cover several aspects of FP programs, including medical indications, or any ethical, social, and legal considerations. Whenever applicable, the first line of action for standard-of-care patients in order to preserve their fertility is gamete and embryo freezing (3, 66, 217–219). For instance, at first, FP in patients undergoing gonadotoxic treatments is either the retrieval of oocytes (preferably mature) and spermatozoa from ejaculation or surgically recovered before cancer therapy treatment, or alternatively, the cryopreservation of embryos after an IVF cycle, as reported in the guidelines applied recently (3, 220). In addition, oocyte cryopreservation has been extensively used and recommended for social freezing reasons, today referred to as “planned oocyte cryopreservation”, and for the establishment of donor egg banks (5, 220, 221).

One of the main and still unresolved issues is the lack of recommendations regarding the accessibility of patients to specific FP programs. The discrepancies between the countries are mainly wrapped around the ethical statements and considerations of these procedures. A paradigm of the above discrepancy is the case of the OTC. This FP process is a prerequisite in hormone-sensitive cancer patients, in prepubertal girls that cannot yet produce oocytes, and in patients that undergo ovariectomy for health and social reasons. For these patients, in 2018–2019, a highly qualified ethical and practice committee (organized by ASRM), granted a green flag to specialized centers to undergo OTC in a clinical standardized environment (3, 222). On the other hand, the European perspective is still reluctant on moving forward. The current European guidelines and country-specific Fertility and Oncology Societies built up a consensus regarding the recommendation and the relative criteria for OTC as a FP process. Nevertheless, the approval of the protocol from the Institutional Review Board of the centers is still mandatory (220, 223, 224). Another issue concerns the use of TTC as a potential procedure in young boys with an increased risk of infertility, and/or with a genetic predisposition for testicular tissue degeneration. In fact, multiple European and American centers offer cryopreservation of immature testicular tissue (to date ~1,033), even though this procedure is still considered experimental (225). This still unsolved matter stresses the need for more clinical trials and follow-ups reporting data on the safety and efficacy of fertility restoration after TTC/TTT.

Notably, the discrepancies regarding the cultures and societies are based mainly on the existing regulation of patients' access to FP programs (226). The vast guidelines (covered mainly by US, European and Australian landscapes) that concern FP programs highlight the progression of legislation and the improvement of the governmental issues regarding access to FP programs. As far as Asia guidelines are concerned, a scarce number of guidelines are coming from Japanese Society, followed by Korean and Chinese Societies (the main different FP programs, divided according to the geographical sources are summarized in Table 2).

**Table 2:** Recommendations for fertility preservations programs.

**FP program**	**Countries**	**Coverage**	**Reference/link**
**Europe**			
Centrum voor Reproductieve Geneeskunde (CRG)	Belgium	Gametes and embryo CPr, OTC, TTC, SSCC	(227)
Edinburgh Fertility and Reproductive Endocrine Center	UK and Northern Ireland	Gametes and embryo CPr, OTC, TTC	(228)
FertiPROTEKT	Germany, Austria, Switzerland	Oocyte and embryo CPr, OTC	(229)
French National Institute of Cancer (INCa) National FP program	France	Gametes and embryo CPr, OTC, TTC	(230)
HUG-CHUV-UKBB FP Pediatric Group	Switzerland	TTC	(231)
IVI Fertility Center	Spain	Oocyte CPr, OTC	(232)
MediPass	Greece	Gametes and embryo CPr	(233)
NORDFERTIL	Sweden, Finland, Norway, Lithuania, Denmark, Estonia, Latvia, Iceland	TTC	(234)
PREFER	Italy	Oocyte CPr, OTC	(235)
SveaFertil	Sweden	OTC	(236)
**America (North, Center and South)**			
Argentine Mastology Society (SAM)—Part of the Oncofertility consortium	Argentina	Oocyte and embryo CPr, OTC	(237)
Instituto Idéia Fertil—Santo André	Brasil	Oocyte CPr	(238)
MAGEE-WOMENS	Pennsylvania	OTC, TTC	(239)
Mayo Clinic—Part of the Oncofertility consortium	Arizona, Florida, Minnesota	Gametes and embryo CPr, OTC, TTC	(240)
MUHC Reproductive Center	Canada	Gametes and embryo CPr, OTC	(241)
**Asia**			
Chinese Maternal and Child Health Association Affiliated Fertility Preservation Professional Committee	China	Oocyte and embryo CPr, OTC	(242)
Center for Fertility Preservation, Kasturba Medical College, Manipal University	India	Gametes and embryo CPr, OTC, TTC	(243)
Japan Society of Clinical Oncology	Japan	Gametes and embryo CPr, OTC, TTC	(244)
Fertility Center of Seoul National University Bundang Hospital	Korea (Republic of)	Oocyte and embryo CPr, OTC	(245)
**Oceania**			
Medical Services Advisory Committee (MSAC)	Australia	Gametes and embryo CPr, OTC, TTC	(246)
**Africa**			
National Research Center (NRC)—Part of the Oncofertility Consortium	Egypt	Gametes and embryo CPr	(247)
Aziza Othmana Hospital of Tunis	Tunisia	Gametes and embryo CPr, OTC	(247)

CPr, cryopreservation; OTC, ovarian tissue cryopreservation; SSCC, spermatogonial stem cell cryopreservation; TTC, testicular tissue cryopreservation.

Finally, it is of paramount importance the commitment of all specialized centers and all experts in the field that are involved in communication with the patients, to promote and suggest, all the necessary information for the options of FP programs, the feasibility, and the safety of the guidelines (248).

## Conclusions and future perspectives

In humans, oocyte and embryo vitrification is a method of FP that is routinely applied. Although vitrification is well-established offering a broad vista in patients undergoing assisted reproductive technologies, its application to other more complex biological materials, such as tissues, is still under investigation. Indeed, oocyte and embryo vitrification have shown increased clinical outcomes. In particular, several IVF centers prefer the “freeze-all” protocol anticipating better IVF outcomes (45, 58). The cryopreservation of embryos, either at the cleavage stage or blastocysts, has already reached the maximum outcome through the vitrification process. However, nowadays, challenges are encountered in developing more standardized, optimal, and successful protocols for the cryopreservation of oocytes and embryos, aiming at maintaining integrity in their structure and function post-thawing. Sperm vitrification is currently applied in samples with reduced sperm concentration and volume, while with increased sperm volumes the choice of SF sounds better. In several cases with a limited number of viable spermatozoa, such as epididymal and testicular samples, the conventional SF method is inappropriate. Nevertheless, single-sperm freezing technology such as the use of empty zona pellucida and the novel SpermVD device gives hope to such patients (249, 250). The existing methods concerning sperm cryopreservation need to be validated in terms of new CPAs or even antioxidants. In addition, the future of sperm cryopreservation might benefit from personalized and individually designed cryopreservation approaches. However, established cryopreservation programs with highly developed protocols and appropriate devices can better support FP procedures and may reshape the cryo-banking landscape of gametes and embryos. Especially in vitrification, there is an urge for established recommendations and guidelines regarding the handling and storing of gametes and embryos. Additionally, the contribution of artificial intelligence and genetic analysis via next-generation sequencing could enhance the success of various cryopreservation programs.

Even if OTC and TTC bio-banking is not routinely applied for patients' FP, the cryopreservation methods are a matter of debate. SF of the OTC cortex is the most applied method, whilst OTV is still clinically uncertain, due to the limited number of babies born. The optimization and standardization of cryopreservation protocols for OTC and OTT are currently ongoing. The health of oncological patients is of pivotal importance in this matter, demanding the development of standard-of-care procedures for improving their safety and fertility lifespan. On the other hand, TTC has been experimentally applied to animal models, but the clinical data reported are not valid. For the optimization of OTV or the standardization of TTC procedures, it is required a deep understanding of freezing/thawing biochemistry in order to improve the safety or to establish the efficiency of the applied techniques. Although TTC is still at an experimental stage and not yet established at a clinical phase, it seems to be a promising FP technique. Once TTC will be refined and validated via appropriate algorithms, it will gain ground for future fertility restoration, something that seems to be an important scientific and clinical achievement for young patients. Most clinical human trials are based on the notion to retain the cryopreserved cells or tissues intact. Concomitantly, it is crucial the development of more accurate freezing protocols and cryostorage systems, which will ensure the correct preservation of tissue materials.

It is of great importance to underline that also the guidelines for gametes, embryos, and tissue cryopreservation have a key role for both providers and patients to decrease the risks linked to an unsafe FP choice and cryostorage. The international reproductive medicine community should make a joint effort to have as a main goal the establishment of global registries regarding FP techniques and their short-/long-term outcomes. This eventually will allow overpassing cultural, societal, and geographical discrepancies and will increase the resonance of counseling, thus empowering the patients' choice for FP programs, and ameliorating the global bioethical harmony.

## Author contributions

Conceptualization: GA. Writing—original draft preparation: SA, VD, and CM. Writing—review and editing: AD, SR, and GA. Supervision: AD and GA. All authors have read and agreed to the version of the manuscript.
